# Effect of different components of triple-H therapy on cerebral perfusion in patients with aneurysmal subarachnoid haemorrhage: a systematic review

**DOI:** 10.1186/cc8886

**Published:** 2010-02-22

**Authors:** Jan W Dankbaar, Arjen JC Slooter, Gabriel JE Rinkel, Irene C van der Schaaf

**Affiliations:** 1 Department of Radiology, University Medical Center Utrecht, Heidelberglaan 100, Utrecht, 3584CX, Netherlands; 2 Department of Intensive Care, University Medical Center Utrecht, Heidelberglaan 100, Utrecht, 3584CX, Netherlands; 3 Department of Neurology (Rudolf Magnus Institute for Neuroscience), University Medical Center Utrecht, Heidelberglaan 100, Utrecht, 3584CX, Netherlands

## Abstract

**Introduction:**

Triple-H therapy and its separate components (hypervolemia, hemodilution, and hypertension) aim to increase cerebral perfusion in subarachnoid haemorrhage (SAH) patients with delayed cerebral ischemia. We systematically reviewed the literature on the effect of triple-H components on cerebral perfusion in SAH patients.

**Methods:**

We searched medical databases to identify all articles until October 2009 (except case reports) on treatment with triple-H components in SAH patients with evaluation of the treatment using cerebral blood flow (CBF in ml/100 g/min) measurement. We summarized study design, patient and intervention characteristics, and calculated differences in mean CBF before and after intervention.

**Results:**

Eleven studies (4 to 51 patients per study) were included (one randomized trial). Hemodilution did not change CBF. One of seven studies on hypervolemia showed statistically significant CBF increase compared to baseline; there was no comparable control group. Two of four studies applying hypertension and one of two applying triple-H showed significant CBF increase, none used a control group. The large heterogeneity in interventions and study populations prohibited meta-analyses.

**Conclusions:**

There is no good evidence from controlled studies for a positive effect of triple-H or its separate components on CBF in SAH patients. In uncontrolled studies, hypertension seems to be more effective in increasing CBF than hemodilution or hypervolemia.

## Introduction

Aneurysmal subarachnoid haemorrhage (SAH) is a subset of stroke that occurs at a relatively young age (median 55 years), and has a high rate of morbidity (25%) and case fatality (35%) [1]. In SAH patients who survive the first days after bleeding, delayed cerebral ischemia (DCI) is an important contributor to poor outcome [2].

Disturbed cerebral autoregulation is often disturbed in SAH patients [3]. In the presence of vasospasm or microthrombosis this may result in decreased cerebral blood flow (CBF) and thereby DCI [3-6]. When autoregulation is affected, CBF becomes dependent on cerebral perfusion pressure and blood viscosity. To increase CBF different combinations of hemodilution, hypervolemia, and hypertension have been used for many years [7]. When all three components are used, the treatment combination is called triple-H [8].

There is no sound evidence for the effectiveness of triple-H or its components on clinical outcome, while triple-H and its components are associated with increased complications and costs [8,9]. To assess the potential of triple-H or its components in improving neurological outcome, knowledge of its effects on its intended substrate, cerebral perfusion, is pivotal.

We aimed to systematically review the literature on the effect of triple-H and its components on CBF in SAH patients and to provide a quantitative summary of this effect.

## Materials and methods

### Search strategy

The Entrez PubMed NIH and EMBASE online medical databases, and the central COCHRANE Controlled Trial Register were searched using the following key terms and MeSH terms: subarachnoid haemorrhage AND (delayed ischemic neurological deficit OR delayed cerebral ischemia OR neurologic deficits OR vasospasm) AND (volume expansion therapy OR hyperdynamic OR hypervolem* OR hemodilution OR hypertens* OR triple-H therapy) AND (cerebral perfusion OR cerebral blood flow). Reference lists from the retrieved reports were checked for completeness. The last search was performed in October 2009.

### Selection criteria

Studies were considered for this review when the investigation was based on human subjects older than 18 years with proven aneurysmal SAH. At least part of the studied population had to be treated with one or more triple-H components and evaluated with a technique measuring CBF. Treatment with triple-H components was considered to be any intervention that aimed to increase blood pressure, to increase circulating blood volume, to cause hemodilution or to result in a combination of these three effects. CBF measurement had to be assessed before and after intervention. Studies from which mean CBF values before and after intervention could not be calculated were excluded. Case reports, reviews and articles that were not obtainable in English, German, French or Dutch were also excluded.

### Data extraction

Two investigators independently assessed eligibility of studies and extracted data by means of a standardized data extraction form. In case of disagreement, both observers reviewed the article in question together until consensus was reached. We extracted data on 1.) study design, 2.) population characteristics, 3.) characteristics of the intervention with triple-H components and 4.) cerebral perfusion. The following items were listed on the standardized extraction form: *Study design: *first year of study, prospective or retrospective design, consecutive series of patient, presence or absence of a control group, and randomization; *Population characteristic: *number of included patients, age, gender, clinical condition (Hunt & Hess grade [10] or World Federation of Neurological Societies (WFNS) [11] score) on admission, and clinical outcome; *Characteristics of the intervention: *type and composition of triple-H components, prophylactic or therapeutic intervention, and intra-cranial and systemic complications; *Cerebral perfusion*: measurement technique, measured part of the brain, time between baseline and follow up CBF measurement (clustered in: < 24 hours, 5 to 7 days, and 12 to 14 days), and difference in CBF between baseline and follow up.

### Analysis

The outcome measurement in this review was the difference in mean CBF between pre- and post-intervention measurements. The 95% confidence intervals (95% CI) of these differences in means were calculated if the sample variance and sample size of the mean pre- and post-intervention measurements were available [12]. The Review Manager software (Review Manager 5, The Nordic Cochrane Centre, Copenhagen, Norway) for preparing and maintaining Cochrane reviews was used for this purpose. If an intervention was done several times, the perfusion measurements around the intervention closest to seven days after SAH were used. Differences in pre- and post-intervention CBF were studied in relation to the time since the start of the intervention (< 24 hours after baseline measurement, 5 to 7 days, or 12 to 14 days after baseline measurement), intention of the intervention (prophylactic or therapeutic (that is, confirmed angiographic vasospasm or symptomatic vasospasm)) and type of intervention (isovolemic hemodilution, hypervolemia, hypertension, or triple-H).

## Results

**Figure 1:**
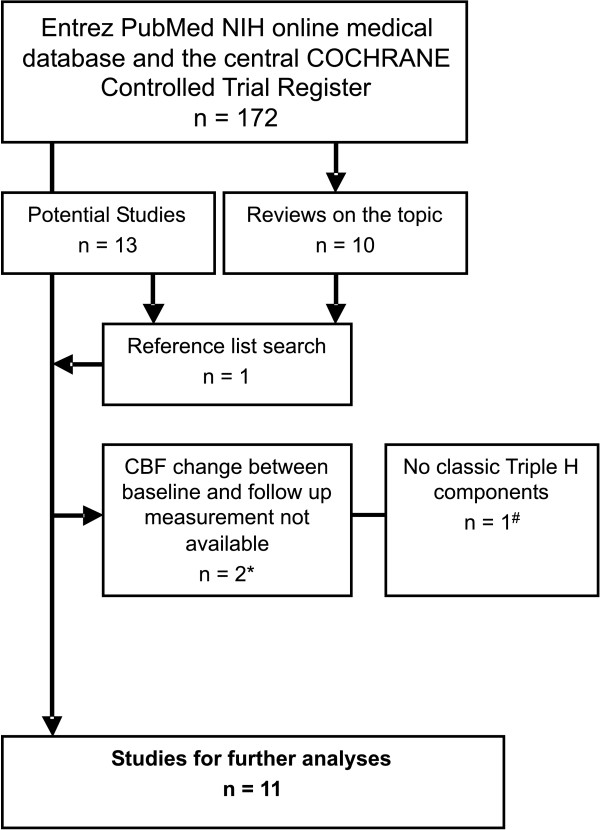
**Flow chart showing the search process for included studies**. Subscript: * Joseph et al [31] and Egge et al [9], # Hadeishi et al [32].

Our literature search resulted in 172 articles. Screening by title and abstract resulted in 13 original studies and 10 review articles on the topic. One more article was identified by reviewing the reference lists of the included studies and the reviews. Of the resulting 14 original studies 11 fulfilled all selection criteria and were used for further analyses (Figure 1).

### Study design and population characteristics

The study design and population characteristics are summarized in Table 1. The 11 included studies were published between 1987 and 2007; eight (73%) of these were prospective. Two studies (18%) [13,14] compared the effect of triple-H components on cerebral perfusion with an independent control group; in one of these interventions allocation was randomized (using hypervolemia as a prophylactic intervention, Table 2), in the other study the intervention and control group differed both in intervention (hypervolemia versus no hypervolemia) and in domain (angiographically confirmed vasospasm versus patients without vasospasm) [14]. Two studies (18%) mentioned that they used a consecutive series of patients [13,15]. The number of included patients varied from 4 to 51 with an average age of 42 to 59 years. In the nine (82%) studies that used the Hunt and Hess scale (H&H) to classify the clinical condition on admission, the median H&H varied between two and four. One study (9%) used the WFNS grading scale including only patients with WFNS 4 and 5. Clinical outcome was described in seven studies (64%), three using the Glasgow outcome scale [16], one using the neurologic outcome by Allen et al [17], and three using not further specified outcome definitions. Eighty to one hundred percent of treated patients showed good recovery or moderate disability.

**Table 1:** Study design and population characteristics:

*Reference*	*Study design*					
	*Intervention type*	*Prophylactic/Therapeutic*	*Prospective*	*Consecutive series*	*Randomized*	*Control group*
Ekelund, 2002 [18]	isovolemic hemodilution or hypervolemic hemodilution	Therapeutic	+	unknown	-	-
Mori, 1995 [14]	hypervolemic hemodilution	Therapeutic	+	unknown	-	+
Yamakami, 1987 [21]	hypervolemia	Prophylactic	+	unknown	-	-
Lennihan, 2000 [13]	hypervolemia	Prophylactic	+	+	+	+
Tseng, 2003 [23]	hypervolemia	Therapeutic	+	unknown	-	-
Jost, 2005 [22]	hypervolemia	Therapeutic	+	-	-	-
Muizelaar, 1986 [25]	hypertension	Therapeutic	unknown	-	-	-
Touho, 1992 [20]	hypertension	Both	unknown	unknown	-	-
Darby, 1994 [24]	hypertension	Therapeutic	-	-	-	-
Origitano, 1990 [15]	Triple-H	Prophylactic	+	+	-	-
Muench, 2007 [19]	Triple-H or hypertension or hypervolemic hemodilution	Prophylactic	+	unknown	-	-
***Reference***	***Population Characteristics***					
	***Nr. Int/noInt***	***Mean age***	***Men***	***Clinical condition on admission: Type, median Int/no Int (range)***	***Good Recovery or moderate Disability: Int/no Int***	***Severe Disability or death: Int/no Int***
Ekelund, 2002 [18]	8/0	42	13%	H&H, 2 (1 to 3)	100%	0%
Mori, 1995 [14]	51/47	56	38%	H&H, 2/2 (1 to 4)	82%/unknown	18%/unknown
Yamakami, 1987 [21]	35/0	51	31%	H&H, ? (1 to 4)	86%	14%
Lennihan, 2000 [13]	41/41	48.5	41%	H&H, 2/2 (1 to 4)	80%/76%	17%/20%
Tseng, 2003 [23]	6/0	50	unknown	WFNS, ? (4 to 5)	unknown	unknown
Jost, 2005 [22]	6/0	49	50%	unknown	unknown	unknown
Muizelaar, 1986 [25]	4/0	44	0%	H&H, 4 (2 to 5)	100%	0%
Touho, 1992 [20]	20/0	55	55%	H&H, 2 (2 to 4)	90%	10%
Darby, 1994 [24]	13/0	59	23%	H&H, 2.5 (1 to 5)	unknown	unknown
Origitano, 1990 [15]	43/0	46	35%	H&H, 2 (1 to 4)	84%	16%
Muench, 2007 [19]	10/0	53	20%	H&H, ? (2 to 5)	unknown	unknown

DCI, delayed cerebral ischemia; H&H, Hunt and Hess grading scale for subarachnoid hemorrhage [10]; Int, Intervention; WFNS, World Federation of Neurological Surgeons score [11]

**Table 2:** Characteristics of the intervention:

*Reference*	*Triple-H components*		*Composition*		*Complications*	
	*type*	*Prophylactic/Therapeutic*	*Intervention group*	*Control group*	*Intracranial* *Int/* *no Int*	*Systemic* *Int/* *no Int*
Ekelund, 2002 [18]	isovolemic hemodilution or hypervolemic hemodilution	Therapeutic	-Isovolemic: Venasection with simultaneous infusion of 70% dextran and 4% albumin in equal volumes -Hypervolemic (after isovolemic): Autotransfusion and infusion of 70% dextran and 4% albumin	-	unknown	unknown
Mori, 1995 [14]	hypervolemic hemodilution	Therapeutic	500 ml human albumin solution, 500 ml low molecular dextran per day	900 ml 10% glycerol per day	0%/unknown	4%/unknown
Yamakami, 1987 [21]	hypervolemia	Prophylactic	500 ml 5% albumin in 30 minutes	-	unknown	unknown
Lennihan, 2000 [13]	hypervolemia	Prophylactic	250 ml 5% albumin in two hours	80 ml 5% dextrose and 0.9% saline in one hour	15%/17%	7%/5%
Tseng, 2003 [23]	hypervolemia	Therapeutic	2 ml/kg 23.5% saline in 20 minutes	-	unknown	unknown
Jost, 2005 [22]	hypervolemia	Therapeutic	15 ml/kg 0.9% saline in one hour	-	unknown	unknown
Muizelaar, 1986 [25]	hypertension	Therapeutic	-Phenylephrine (mean MAP increase of 33 mmHg) -hypervolemia with Ht around 32%	-	0%	0%
Touho, 1992 [20]	hypertension	Both	Continuous infusion of dopamine 7 to 15 μg/kg/min (mean MAP increase of 22 mmHg)	-	unknown	unknown
Darby, 1994 [24]	hypertension	Therapeutic	dopamine 6.4 to 20 μg/kg/min (mean MAP increase of 21 mmHg)	-	unknown	unknown
Origitano, 1990 [15]	Triple-H	Prophylactic	-Venasection to Ht of 30 in increments of 150 to 250 ml every eight hours within 12 to 24 hours -infusion of 250 to 500 ml 5% albumin every six hours -dopamine or labetolol (mean MAP increase not written)	-	0%	9%
Muench, 2007 [19]	Triple-H or hypertension or hypervolemic hemodilution	Prophylactic	-norepinephrine to raise MAP above 130 mmHg (mean MAP increase not written) -1,000 ml hydroxyethyl-starch and 1,000 to 3,000 ml crystalloids	-	unknown	unknown

Ht, hematocrit; Int, Intervention

### Characteristics of the Intervention

The details of the intervention are summarized in Table 2. One study used isovolemic hemodilution, seven used hypervolemia (three of these with hemodilution), four used induced hypertension, and two used triple-H components. Two studies applied several triple-H components in succession within the same patient and compared their effect on CBF [18,19]. Four (36%) studies applied the intervention in SAH patients without DCI or vasospasm (prophylactically), six (55%) in SAH patients with DCI or vasospasm (therapeutically), and one (9%) applied the intervention both therapeutically and prophylactically. To achieve isovolemic hemodilution, venasection was simultaneously performed with infusion of 70% dextran and 4% albumin. To achieve hypervolemia a 4 to 5% albumin solution was most commonly used. The total volume of administered fluids was not always provided in the study reports; in those who provided this item, it varied between 250 to 4,000 ml per day. To induce hypertension either phenylephrine or dopamine was used. This resulted in an average increase in mean arterial pressure (MAP) of 21 to 33 mmHg. Four studies mentioned the occurrence of complications during intervention with triple-H components, with systemic complications (congestive heart failure, pulmonary oedema, diabetes insipidus, electrolyte disturbances) being less frequently present (0 to 9%) than intracranial complications (cerebral oedema, 0 to 17%). None of the complications were fatal.

### Cerebral perfusion

**Figure 2:**
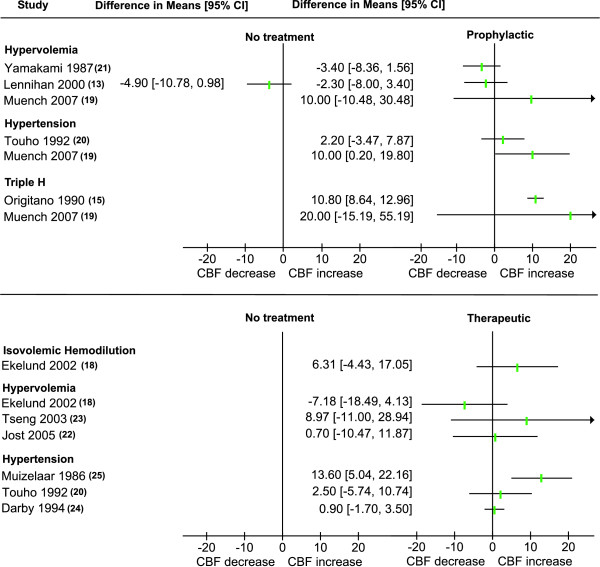
**Mean CBF (ml/100 g/min) difference between start of intervention and follow-up within 24 hours**.

**Figure 3:**
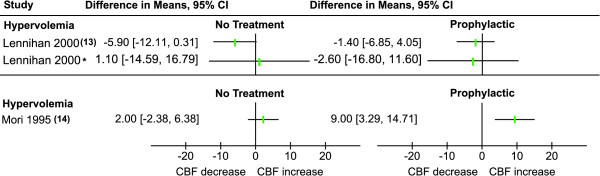
Mean CBF difference between start of intervention and follow-up within 5-7 days and 12-14* days.

Cerebral perfusion measurement details are summarized in Table 3. Different perfusion measurement techniques were used: five (45%) studies used an external scintillation counter (e.s.c.) technique, one (9%) used single photon emission computed tomography (SPECT), three (27%) used Xenon-CT (XeCT), one used (9%) PET and one (9%) study thermal diffusion microprobes (validated by XeCT). Four (36%) studies did not report whole brain perfusion measurements, but only measurements from the hemisphere ipsilateral to craniotomy or in the flow territory distal to the aneurysm [14,19-21]. Nine (82%) studies measured CBF within 24 hours after the start of the intervention and two at a later time. These two studies both measured after five to seven days and one also after 12 to 14 days. Differences in mean CBF before and after intervention with their 95% confidence intervals are plotted in Figures 2 and 3. Weighted total effects could not be calculated due to the large heterogeneity in the used intervention, the studied populations and the applied methods.

**Table 3:** Cerebral perfusion measurement

*Reference*	*Triple-H components*	*Prophylactic/Therapeutic*	*CBF Technique*	*Measuring location*	*Timing after Intervention*
Ekelund, 2002 [18]	isovolemic hemodilution or hypervolemic hemodilution	Therapeutic	^133^Xe SPECT	Whole brain	< 24 hours
Mori, 1995 [14]	hypervolemic hemodilution	Therapeutic	^123^I-IMP e.s.c.	Ipsilateral to craniotomy	5 to 7 days
Yamakami, 1987 [21]	hypervolemia	Prophylactic	^133^Xe e.s.c.	Ipsilateral to craniotomy	< 24 hours
Lennihan, 2000 [13]	hypervolemia	Prophylactic	^133^Xe e.s.c.	Whole brain	< 24 hours 5 to 7 days 12 to 14 days
Tseng, 2003 [23]	hypervolemia	Therapeutic	XeCT	Whole brain	< 24 hours
Jost, 2005 [22]	hypervolemia	Therapeutic	PET	Whole brain	< 24 hours
Muizelaar, 1986 [25]	hypertension	Therapeutic	^133^Xe e.s.c.	Whole brain	< 24 hours
Touho, 1992 [20]	hypertension	Both	XeCT	Ipsilateral to craniotomy	< 24 hours
Darby, 1994 [24]	hypertension	Therapeutic	XeCT	Whole brain	< 24 hours
Origitano, 1990 [15]	Triple-H	Prophylactic	^133^Xe e.s.c.	Whole brain	< 24 hours
Muench, 2007 [19]	Triple-H or hypertension or hypervolemic hemodilution	Prophylactic	thermal diffusion microprobe	in flow territory distal to aneurysm	< 24 hours

CBF, cerebral blood flow; e.s.c., external scintillation counter.

#### Short term (within 24 hours) effects of prophylactic use of triple-H components

When compared to baseline measurement, hypervolemia led to a non-significant CBF decrease in two studies [13,21] and a non-significant CBF increase in one study [19]. Hypertension was associated with an increase in CBF in two studies, this was statistically significant in one (increase of 10 ml/100 gr/min) [19]; triple-H led to CBF increase in two studies [15,19], this was statistically significant in one (increase of 11 ml/100 gr/min) [15]. The study that compared hypervolemia to a control group found no statistically significant difference between both groups [13].

#### Short term (within 24 hours) effects of therapeutic use of triple-H components

Isovolemic hemodilution resulted in a non-significant CBF increase [18]. Hypervolemia was associated with a non-significant increase in two studies [22,23] and decrease in one [18], and hypertension resulted in a CBF increase in three studies [20,24,25] which was significant in one (increase of 13 ml/100 gr/min) [25]. All these changes were compared to baseline values. None of these studies compared the effects to a control group.

#### Long term (5 to 7 days and 12 to 14 days) effects of triple-H components

When compared to baseline measurement, prophylactic hypervolemia resulted in a non-significant CBF decrease in the intervention group both after 5 to 7 days and 12 to 14 days, in the control group a non-significant decrease after 5 to 7 days and increase after 12 to 14 days was seen [13]. Therapeutic hypervolemia resulted in a significant CBF increase (mean increase of 9 ml/100 gr/min) compared to baseline values; the untreated control group without vasospasm showed no significant CBF increase [14].

## Discussion

Triple-H and its separate components aim to increase cerebral perfusion and thereby improve outcome. Given the lack of randomized clinical trials on triple-H and clinical outcome, we evaluated the evidence of the effect of triple-H components on CBF. Due to the large heterogeneity in study design, CBF measurement, and composition of triple-H components, it was not possible to perform a meta-analysis of treatment effects of the included studies. We therefore assessed the results of the individual studies separately.

There is no good evidence that isovolemic hemodilution or hypervolemia improve CBF in the initial days. One study found a remote effect of hypervolemia compared to baseline, but did not use a proper control group [14]. Induction of hypertension, alone or combined with hypervolemia did improve CBF compared to baseline levels in three separate studies. It could be concluded that this component is the most promising. However, without a control group within the same population, one can not be sure that the observed changes in CBF do not just reflect the natural course of cerebral perfusion after SAH.

Apart from lack of properly controlled studies, there are other potential drawbacks of the presented evidence from the literature. First, we are likely dealing with publication bias since positive studies have a greater chance of being reported. Second, several of the included studies had small sample sizes (< 10 patients) and are therefore likely to represent a selection of successful cases. Third, there was a large heterogeneity in methods of CBF measurement making generalized conclusions and meta-analyses impossible. Although the used CBF measurement techniques have been validated [26], small changes in CBF may not be picked up equally well by the different techniques. Furthermore, in some studies CBF was not measured in the entire brain but only in the separate hemispheres. In these studies we chose to analyze the CBF change in the hemisphere ipsilateral to craniotomy or in the flow territory distal to the aneurysm, since the risk of ischemia is highest in that region [27]. The changes induced by triple-H therapy are likely to be larger in that part of the brain, compared to the measurements in both hemispheres combined. Another issue is the composition of triple-H. The different triple-H components aim to influence perfusion pressure and blood viscosity in order to increase CBF [28]. Whether induction of hypertension is successful in terms of raising blood pressure is easily controlled, although there is no consensus on the degree and duration of induced hypertension. The discrepancies in effects on CBF within the different studies on hypertension may therefore be explained at least in part by different hypertension strategies. Whether strategies aiming for hemodilution and hypervolemia actually achieve these effects is unsure [29,30]. Triple-H combines hypertension, hemodilution and hypervolemia, and should theoretically result in the largest CBF increase, but we could not confirm this in this review.

We acknowledge the fact that an increase in CBF does not imply that the outcome of SAH improves. First, this increase may only be transient or not sufficient to prevent ischemia and infarction. Second, oxygen delivery may not be increased despite the increase in CBF. This has been described in a study on the effect of hypervolemia on brain oxygenation and is most likely caused by hemodilution resulting from the volume expansion [19]. However, since an increase in CBF is the mechanism by which triple-H and its components should improve outcome, explanatory (phase II) randomized trials showing an increase in CBF measurements from triple-H or its components are crucial before large effectiveness trials are undertaken. The estimated sample size needed for such a phase II trial to properly analyze the effect of triple-H on CBF is not too large. The data in this review show that the size of significant CBF changes in the presented studies were approximately 10 ml/100 gr/min and that the mean standard deviation (based on the confidence intervals in Figure 2) for CBF differences was about 18 ml/100 gr/min. To detect an effect size of 10 ml/100 gr/min difference in CBF change between treated and untreated DCI patients (with a standard deviation of 18 ml/100 gr/min) 104 patients (52 in each group) are needed to obtain a statistical power of 80% with an α of 0.05.

## Conclusions

This review of the literature gives a quantitative summary of the effect of triple-H and its components on CBF, the intended substrate of this intervention. We showed that there is no good evidence that CBF improves due to the intervention. From all components of triple-H, induced hypertension seems to be the most promising. A pivotal first step is to conduct a randomized controlled trial in SAH patients with DCI on the effect of induced hypertension on CBF.

## Key messages

• There is no evidence from controlled trials that triple-H or its separate components increase CBF in SAH patients.

• Of all triple-H components induced hypertension has the most consistent CBF increasing effect, if comparing baseline CBF to follow-up measurements.

• There is no consensus on how triple-H or its separate component should be applied.

## Abbreviations

CBF: cerebral blood flow; CI: confidence interval; DCI: delayed cerebral ischemia; e.s.c.: internal scintillation counter; MAP: mean arterial pressure; PET: positron emission tomography; SAH: subarachnoid haemorrhage; SPECT: single photon emission computed tomography; triple-H: hemodilution, hypervolemia and hypertension; WFNS: world federation neurological surgeons; XeCT: Xenon-CT

## Competing interests

The authors declare that they have no competing interests.

## Authors' contributions

JWD designed the study, collected the data, performed the statistical analysis, and drafted the manuscript. AJCS helped design the study, checked the data collection and the statistical analysis, and helped to draft the manuscript. GJER helped design the study and helped to draft the manuscript. ICvdS coordinated the study, collected the data, checked the statistical analysis and helped to draft the manuscript. All authors read and approved the final manuscript.
